# Chronic Idiopathic Recurrent Orbital Myositis in a Patient With Concomitant Late-Onset Hashimoto’s Thyroid Eye Disease

**DOI:** 10.7759/cureus.97629

**Published:** 2025-11-24

**Authors:** Palvi Bhardwaj, Alberto Chierigo, Alice Mason, Ma'en Al-Mrayat, Daniele Lorenzano

**Affiliations:** 1 Oculoplastic and Orbital Service – Eye Unit, Southampton General Hospital, University Hospital Southampton NHS Foundation Trust, Southampton, GBR; 2 Rheumatology, Southampton General Hospital, University Hospital Southampton NHS Foundation Trust, Southampton, GBR; 3 Endocrinology, Southampton General Hospital, University Hospital Southampton NHS Foundation Trust, Southampton, GBR

**Keywords:** orbital myositis, rituximab, ted, thyroid eye disease, hashimoto’s thyroiditis

## Abstract

A 38-year-old female presented to a tertiary centre with a nine-year history of chronic recurrent isolated lateral rectus orbital myositis (OM). Initially, her condition responded to short courses of oral corticosteroids and orbital steroid injections, but it later became refractory, with an inability to taper off steroids. Concurrently, the patient developed a low-grade clinical activity score for bilateral thyroid eye disease (TED) due to Hashimoto’s thyroiditis (HT). Although the active phase of TED resolved without significant sequelae, she continued to experience unilateral pain and extraocular muscle (EOM) motility restriction consistent with lateral rectus myositis. This report describes a rare case of chronic recurrent idiopathic OM overlapping with a concomitant bilateral TED associated with HT, which was successfully managed with rituximab immunosuppression.

## Introduction

Orbital myositis (OM) is a well-recognised inflammatory condition affecting one or more extraocular muscles (EOM) within the orbit. Although relatively rare, it is the second most common cause of EOM inflammation after thyroid eye disease (TED) [1]. The most frequent presentation is idiopathic acute-onset OM in young adult women, typically manifesting as painful diplopia involving a single EOM. While OM can occur as a chronic and recurrent idiopathic condition, it may also be associated with specific autoimmune, infectious, or inflammatory disorders, with numerous atypical variants reported in the literature [2,3]. OM has also been shown to occur in association with other autoimmune diseases [4].

TED, also known as Graves’ orbitopathy, is an autoimmune, multifactorial inflammatory disease involving the eye, EOMs, and the soft tissues within the orbit. While it is most commonly linked to hyperthyroidism due to Graves’ disease [5], TED has also been reported, albeit rarely, in patients with hypothyroidism, euthyroidism, and Hashimoto’s thyroiditis (HT). [6,7]. It has an incidence of 0.54-0.9 cases/100,000/year in men and 2.67-3.3 cases/100,000/year in women [8]. The pathophysiology of TED remains incompletely understood, though a widely accepted hypothesis involves an autoimmune response against antigens shared by the thyroid and orbit. The thyrotropin (TSH) receptor and the insulin-like growth factor-1 (IGF-1) receptor are two known targets of this autoimmune disease, and studies have shown their role in both the onset and treatment of TED [9,10]. Antibodies against the TSH receptor were also found in patients with HT and thyroid-associated ophthalmopathy [11]. In this report, we describe a rare case of a chronic recurrent idiopathic OM worsened by a concomitant bilateral TED presentation driven by HT.

This article was previously presented as a poster at the European Society of Ophthalmic Plastic Surgery (ESOPRS) and British Oculoplastic Surgery Society (BOPSS) meetings in 2023.

## Case presentation

**Figure 1:**
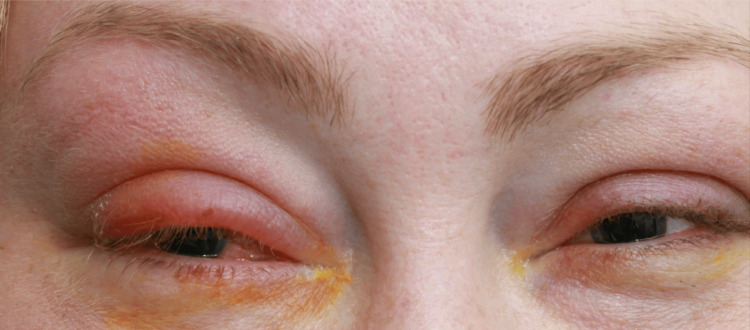
Right upper lid oedema, erythema, and conjunctival injection

A 38-year-old female presented in 2009 with ocular pain, swelling (Figure 1), and diplopia. MRI of the orbits revealed swelling and enhancement of the right lateral rectus muscle, consistent with OM. She had no other symptoms of myositis elsewhere and no other symptoms suggestive of a connective tissue disease. Her antinuclear antibodies (ANA), extractable nuclear antigen (ENA) antibodies, and extended myositis panel were negative, leaving the underlying cause unclear (ds-DNA: 2 IU/mL (reference range (RR): <10 IU/mL), proteinase 3 antibody: 0.3 IU/mL (RR: <2 IU/mL), myeloperoxidase antibody: 0.1 IU/mL (RR: <5 IU/mL), neutrophil anti-MPO antibodies: <0.2 (RR: <3.5 IU/mL), neutrophil anti-PR3 antibody: <0.2 (RR: <2 IU/mL), and immunoglobulin G4: 0.24 g/L (RR: <1.3 g/L)). She had no past medical history but was later diagnosed with hypothyroidism with positive thyroid peroxidase (TPO) antibodies. Throughout the 11 years since diagnosis, her TED had been well controlled, and she had had normal free T3/T4 and thyroid-stimulating hormone (TSH) levels. It was felt that TED was unlikely to present with isolated involvement of a single muscle.

**Figure 2:**
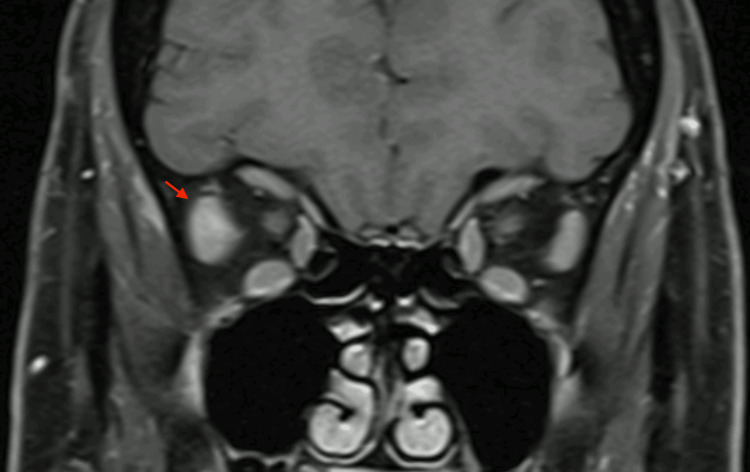
Coronal view of T1-weighted MRI test The MRI shows abnormal swelling of the right lateral rectus belly (arrow) MRI: magnetic resonance imaging

The patient was initially treated with 40 mg of oral prednisolone, tapered over six weeks, which led to complete resolution of her symptoms. She experienced her first relapse in 2013, successfully managed with a 12-month tapering course of prednisolone. During her third relapse in 2019, corticosteroids were again administered; however, she became steroid-dependent, requiring 60 mg of oral prednisolone. She was therefore referred to rheumatology for consideration of steroid-sparing therapy. Between 2013 and 2019, MRI demonstrated progressive enlargement of the right lateral rectus muscle with reduction of the intra-orbital space (Figure 2).

**Figure 3:**
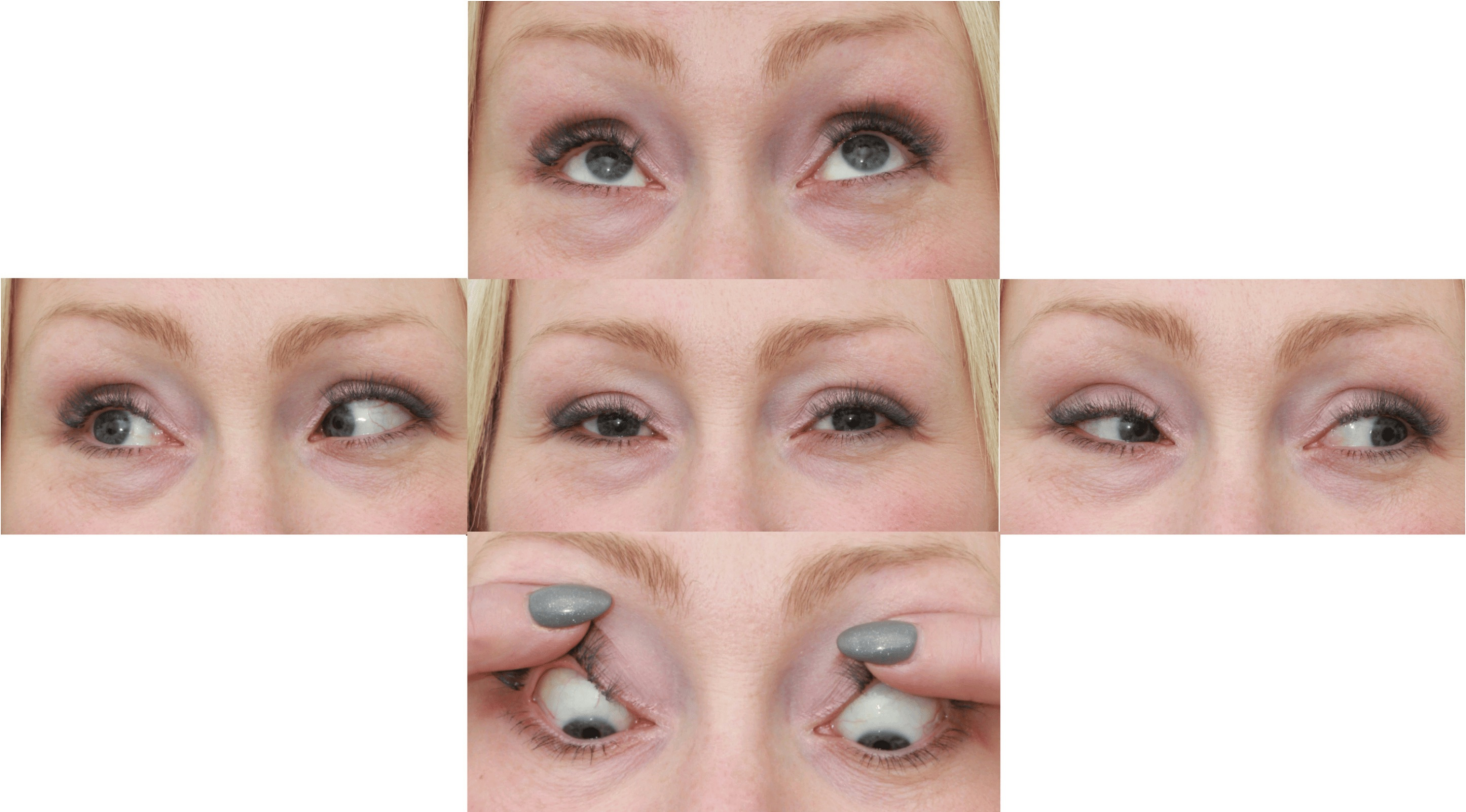
External photographs showing residual restriction in abduction of the right eye

Under the care of rheumatology, a repeat connective tissue disorder and myositis screen was performed, which returned normal results. Despite minimal sequelae of TED, the patient continued to experience unilateral persistent pain and diplopia. A steroid-sparing agent (azathioprine) was trialled with little effect, and attempts to taper prednisolone below 30 mg daily were unsuccessful. Intra-orbital steroid injections were also tried with minimal benefit. A course of rituximab was started in June 2021, after which corticosteroids were successfully discontinued. She subsequently received a second dose of rituximab and has remained free of active symptoms, though she retains some extraocular motility restriction due to the chronic relapsing course (Figure 3). This has been managed with a Fresnel prism for symptomatic relief.

## Discussion

Several different orbital conditions present with a picture of inflammation. This may be systemic or localised, present acutely or chronically, and occur focally or diffusely in the orbit. Common causes include thyroid orbitopathy, orbital cellulitis, non-specific orbital inflammatory disease, and specific orbital inflammatory disorders. The differential diagnosis for orbital inflammation spans infectious, inflammatory, and neoplastic processes [12-14].

Non-specific orbital inflammation (NSOI) was first described in 1905 by Felix Victor Birch-Hirschfeld, a German pathologist with a particular interest in infectious diseases, especially tuberculosis [15]. It is a benign, non-infectious inflammatory process of the orbit characterised by a polymorphous lymphoid infiltrate with varying degrees of fibrosis, without a known local or systemic cause. Swamy et al., in 2007, reviewed 24 patients who had been biopsied with non-specific orbital inflammation; they found that the lacrimal gland was affected in 54.2%, EOM in 50%, orbital fat in 75%, optic nerve in 20.8%, sclera in 4.2% and other structures within the orbit in 8.3% of the time [13]. Idiopathic OM is considered a subtype of idiopathic orbital inflammation with EOM being primarily impacted by the inflammatory process, leading to an acute, painful clinical presentation associated with diplopia and periorbital swelling [13]. It most commonly involves a single EOM, typically the medial or lateral rectus [16].

OM can be a debilitating condition causing diplopia and pain, as illustrated by the case of a 38-year-old woman who has been unable to work for the past year due to her symptoms. It can affect single muscles or multiple muscles and may be unilateral or bilateral [2,17]. The leading differential diagnosis is TED. However, TED does not usually cause an isolated myopathy; it is usually painless on ocular movements and slowly progressive, and as such was felt unlikely to be the underlying pathology in this case. Moreover, the lateral rectus is the least frequently affected muscle in TED, likely due to a different embryogenesis, with the lateral rectus originating from the unsegmented paraxial head mesoderm, and the other EOM developing from prechordal head mesoderm [16,18]. Finally, the frequency of TED is much less in HT than in Graves’ disease, with roughly 5% of patients with Hashimoto’s developing TED, whereas this occurs in 25-50% of patients with Graves’ disease [19]. 

Another important differential diagnosis is IgG-4-related disease. This can lead to lateral rectus inflammation, but it is usually characterised by bilateral involvement of multiple EOMs and other orbital structures, such as the lacrimal gland, the infraorbital nerve, the frontal nerve, as well as thickening of the sinus mucosa [16]. Our case did not fit with this presentation. The localised involvement of a single EOM and the good response to steroids make a diagnosis of an infectious disease quite unlikely. Salient features from current literature on idiopathic orbital inflammation, including myositis, suggest that 75% of patients are found to have a good response to corticosteroids [2,3,13]. Second-line treatments include radiotherapy, methotrexate/azathioprine, and other biologic agents [17].

A case report in 2014 involving 10 patients with OM refractory to steroids and at least one other immunosuppressant demonstrated that rituximab was safe and effective, with 7/10 patients noting improvement of their symptoms [20]. Out of those seven patients, four had been on steroids at the induction of rituximab, and all patients were able to reduce their steroid dose. The patients in this trial received two initial doses of rituximab and were permitted to have a further dose at 24 weeks if there was a recurrence of symptoms. Four out of the seven patients required a further infusion after 24 weeks. 

We identified two additional case reports from 2008 and 2012 describing the use of rituximab in OM, both demonstrating a favorable response. Both patients were unable to wean from corticosteroids and had tried other disease-modifying antirheumatic drugs (DMARDs) such as methotrexate. Other reports of immunomodulatory therapy include a case involving two patients treated with adalimumab, which enabled steroid reduction and maintained good outcomes for at least nine months. Rituximab is usually well-tolerated, although adverse effects have been reported, including infusion reactions, infections, malignancies, thromboembolism, myocardial infarction, stroke, nausea, diarrhoea, cytopenia, myalgia, and rashes.

This steroid-refractory case of OM highlights the need to investigate for other causes of EOM enlargement, including but not limited to repeat serum tests to look for other causes of myositis, thyroid antibodies, as well as further imaging to characterise the pattern of muscle involvement. Unilateral involvement of a single EOM is highly suggestive of OM, and this diagnosis was confirmed through multidisciplinary team discussions, where it was determined that muscle biopsy was unnecessary. Biopsy carries inherent risks and may exacerbate already debilitating symptoms such as diplopia.

## Conclusions

TED is most commonly associated with Graves’ disease and is less frequently seen in hypothyroidism-related autoimmune HT, as in this patient. In fact, the patient tested negative for thyroid receptor antibodies but positive for TPO antibodies, demonstrating a classic pattern for HT. Although cases of TED arising from HT have been reported, to our knowledge, this is the first reported case of TED driven by HT overlapping a long course of a unilateral OM successfully treated with immunosuppressive rituximab treatment. This case raises considerations for a potential shift in treatment strategy. Further research is needed to systematically evaluate the response to rituximab in OM and TED associated with HT. We recommend early intervention with immunomodulatory therapy in refractory cases to prevent muscle fibrosis and restrictive strabismus, as demonstrated in this patient.
